# Chemical composition and biological activity of star anise *Illicium verum* extracts against maize weevil, *Sitophilus zeamais* adults

**DOI:** 10.1093/jis/14.1.80

**Published:** 2014-06-22

**Authors:** Linlin Wei, Rimao Hua, Maoye Li, Yanzhang Huang, Shiguang Li, Yujie He, Zonghai Shen

**Affiliations:** 1 College of Plant Protection, Anhui Agricultural University, Hefei 230036, China; 2 College of Resources and Environment, Anhui Agricultural University, Hefei 230036, China; 3 Anhui Zhonggu State Grain Reserve Warehouse, Hefei 231131, China

**Keywords:** contact toxicity, gas chromatography-mass spectrometry, repellency

## Abstract

This study aims to develop eco-friendly botanical pesticides. Dried fruits of star anise ( *Illicium verum* Hook.f. (Austrobaileyales: Schisandraceae)) were extracted with methyl alcohol (MA), ethyl acetate (EA), and petroleum ether (PE) at 25°C. The constituents were determined by gas chromatography-mass spectrometry, and the repellency and contact toxicity of the extracts against *Sitophilus zeamais* Motschulsky (Coleoptera: Curculionidae) adults were tested. Forty-four compounds, whose concentrations were more than 0.2%, were separated and identified from the MA, EA, and PE extracts. The extraction yields of trans-anethole, the most abundant biologically active compound in *I. verum* , were 9.7%, 7.5%, and 10.1% in the MA, EA, and PE extracts, respectively. Repellency increased with increasing extract dose. The average repellency rate of the extracts against *S. zeamais* adults peaked at 125.79 µg/cm ^2^ 72 hr after treatment. The percentage repellency of the EA extract reached 76.9%, making it a class IV repellent. Contact toxicity assays showed average mortalities of 85.4% (MA), 94.5% (EA), and 91.1% (PE). The EA extract had the lowest median lethal dose, at 21.2 µg/cm ^2^ 72 hr after treatment. The results suggest that *I. verum* fruit extracts and trans-anethole can potentially be developed as a grain protectant to control stored-product insect pests. Other active constituents in the EA extract merit further research.

## Introduction

*Sitophilus zeamais* Motschulsky (Coleoptera, Curculionidae) is one of the major pests of stored products. It infests rice, wheat, corn, lotus seed, *Rhizoma gastrodiae,* and Chinese herbs, causing heavy losses of both quantity and quality of stored products worldwide. It attacks intact grains and is quite destructive, as its larvae and adults feed and develop inside the kernel (Lopez et al. 2008; Ojo and Omoloye 2012 ).

Control of *S. zeamais* populations around the world is primarily dependent on continued applications of synthetic insecticides ( Liu et al. 2007 ). Although effective, the long repeated use of synthetic pesticides has led many insect pests, including *S. zeamais,* to develop pesticide resistance ( Isman 2006 ) and pest resurgence. This phenomenon contributes to the vicious cycle of increasing doses, increasing application costs, and enhancing resistance. Moreover, chemical pesticide residues have exceeded acceptable limits in food and have polluted the environment, directly affecting the health of users and consumers. Therefore, investigating and using effective low- to nontoxic alternatives to synthetic pesticides to control stored-product pests is essential in ensuring food safety and human health.

Botanical pesticides have many advantages over synthetic pesticides, such as high selectivity, low or non-toxicity to nontarget organisms and the environment, rapid degradation, low residue, local availability, little cross-resistance due to their natural complex agents, and novel modes of action against insects ( Isman 2006 , 2008; Kudom et al. 2011 ; Lv et al. 2012 ; Ladhari et al. 2013 ) Therefore, increasing attention has been given to natural products from plants constituting a rich source of bioactive chemicals. Fennel, black pepper, menthol, camphor, eucalyptus oil, pyrethrum, and other vegetable matter have different degrees of effects on *S. zeamais* ( Ukeh et al. 2009 ; Li et al. 2010 ).

*Illicium verum* Hook. f (Austrobaileyales: Schisandraceae), a fruit commonly known as star anise, is native to southwest China and Vietnam and is mainly distributed in the tropical and subtropical areas of Asia. *Illicium verum* was considered as one of the things “both food and medicine” by the Ministry of Health of the People's Republic of China (2002), implying its low or non-toxicity to humans. At present, the research focus on *I. verum* has been mainly on food and medical fields ( Ohira et al. 2009 ). The fruits are commonly used as an ingredient of the traditional “five-spice” powder of Chinese cooking, and the essential oil of *I. verum* can be used as a flavoring. The extraction from *I. verum* has carminative, stomachic, stimulant, and diuretic properties, and is used as a pharmaceutical supplement ( Lee et al. 2003 ). Shikimic acid extracted from *I. verum* is one of the main ingredients in the antiviral drug Tamiflu used to fight avian influenza ( Ohira et al. 2009 ). It has also been reported to possess antimicrobial ( Singh et al. 2006 ; Yang et al. 2010 ) andanti-oxidative properties ( Padmashree et al. 2007 ) as well as significant anticancer potential ( Shu et al. 2010 ).

In a previous screening program for new agrochemicals from Chinese medicinal herbs, *I. verum* powder has been shown to possess insecticidal activity against *S. zeamais* and *Cryptolestes pusillus* Schönherr ( Li et al. 2013 ). Other studies have indicated that the essential oil of *I. verum* has repellent and fumigant actions on *S. Zeamais, Blattella germanica* ( Chang and Ahn 2002 ), *Lasioderma serricorne, Sitophilus oryzae, Callosobruchus chinensis* ( Kim et al. 2003a , b), and *Aedes aegypti* ( Dana and Wej 2006 ) *.* However, detailed studies on the biological activities of *I. verum* extracts against *S. zeamais* are lacking. Thus, in the current study, compounds of *I. verum* were extracted from the dried fruits with three types of organic solvents. The extract constituents were then determined by gas chromatography-mass spectrometry (GC-MS), and the repellency and contact toxicity of the extracts against *S. zeamais* adults were evaluated. The present study aims to provide additional data in support of its utilization and development as a new green storage protectant for control of stored-product insect pests.

## Materials and Methods

### Insects

Adults of *S. zeamais* were originally collected from rice stores at Anhui Zhonggu State Grain Storage (Hefei, Anhui, China). The species were kept in the insectarium of the Plant Protection College of Anhui Agricultural University for more than five years. The insects were reared on whole wheat grains that were disinfected in the oven for 2 hr at 100°C; moisture content was adjusted to 13 ± 1%. The insects were placed in a glass jar (250 mL) containing 150 g of sterilized wheat grains (covered with a 60-mesh cloth) without exposure to any insecticide and then incubated at a temperature of 28 ± 1°C, a relative humidity of 70 ± 5%, and a 12:12 L:D photoperiod. The adults were left for seven days for oviposition and then sieved out. Adults (seven to 14 days old) were then used for the succeeding experiments.

### Plant extract preparation

Fruits of *I. verum* were produced in Guangxi, China, but were purchased from a local supermarket in Hefei, China. *Illicium verum* fruits were dried in an oven (GRX-9071B; www.chem17.com ) for two days at 40°C, ground to powder using an electric grinding mill (QE-100; Zhejiang, www.wyyili.com ), and then sifted through a 40-mesh sieve. The *I. verum* dry powder (150 g) was placed in a 1.0 L round-bottomed flask. Methyl alcohol (MA; polarity, 5.1; highly polar), ethyl acetate (EA; polarity, 4.4; weakly polar), and petroleum ether (PE; boiling point range, 60°C to 90°C; polarity, 0.0; non-polar) were respectively added in a ratio of 1:5 (w/v) at room temperature (25°C), incubated in the dark for 48 hr, and then filtered (Whatman No. 2). The samples were leached twice using the same procedure. The final filtrates were collected for each solvent to obtain the respective crude extracts. The combined filtrate was concentrated to dryness using a vacuum rotary evaporator (Buchi Rotavapor R-124; www.buchi.com ) and weighed using an electronic balance (FA2104; www.testmart.cn ). The extraction rates were then calculated. All samples were stored in air-tight brown bottles at 4°C in a refrigerator for use in the subsequent experiments.

### GC-MS

The composition of the extracts was investigated by GC-MS performed on a Varian Saturn 2200 GC system equipped with a capillary column with CP-Sil8CB-MS (30 m × 0.25 mm × 0.25 um) (Agilent Technologies, www.chem.agilent.com ). The GC settings were as follows: the initial oven temperature was held at 50°C for 3 min and ramped at 20 °C/min to 120°C without reserve and then ramped at 10°C/min to 250°C for 5 min. The temperatures of the injector, interface, and gasification chamber were maintained at 260, 250, and 250°C, respectively. The samples (0.4 μL) were injected neat, with a split ratio of 1:100. The carrier gas was helium (99.999%). Mass spectra were obtained at 70 eV by electron impact ionization source. The temperatures of the interface and iron trap were 250 and 150°C. The electron multiplier voltage was 2.4 kV. The mass range analyzed was 20 amu to 650 amu. Most constituents were identified using GC by comparison of their retention indices based on literature data and MS data obtained from Saturn and NIST libraries. Component relative percentages were calculated based on GC peak areas without using correction factors ( Gholivand et al. 2009 ; Chu et al. 2011 ).

### Bioassays

#### Repellency assay.

The repellent activities of the extracts were assessed using a previously described filter-paper drug-film area preference method ( Tapondjou et al. 2005 ) with slight modifications. Test areas consisted of 9 cm Whatman No. 2 filter paper cut in half (31.8 cm ^2^ ). Each extract (50.00 mg) was diluted with acetone to produce the following concentrations: 0.50, 1.00, 2.00, 4.00, and 8.00 mg/mL. Each diluent (0.5 mL) was uniformly dropped on the half filter paper disc with a pipette, corresponding to doses of 7.86, 15.72, 31.45, 62.89, and 125.79 µg/cm ^2^ , whereas the other half of the paper was treated with 0.5 mL acetone alone as a control. The treated and control half discs were air dried at room temperature for 5 min to evaporate the solvents. The treated and untreated halves were attached to their corresponding halves using adhesive tape and then placed in 9 cm glass Petri dishes. The bottom of the dish was daubed with solid glue to prevent insects from going under the filter paper. Thirty mixed-sex *S. zeamais* adults were released at the center of each filter paper disc without food. Glass loops (8.8 cm i.d. × 6.0 cm) coated with polytetrafluoroethylene were then set on the interior walls of the Petri dishes to prevent insects from climbing upward. The dishes were kept in continual darkness. Each treatment was repeated three times. The numbers of *S. zeamais* present on the treated and untreated portions of the experimental paper halves were recorded after 24, 48, and 72 hr of exposure.

#### Contact toxicity assay.

The Petri dish drug-film contact method was adopted in the contact toxicity examination with some adjustments ( Huang et al. 2011 ). Each extract (50.00 mg) was respectively diluted with acetone to the following concentrations: 0.50, 1.00, 2.00, 4.00, and 8.00 mg/mL. Subsequently, 1.00 mL of each solution was poured into clean Petri dishes (9.0 cm i.d. × 1.0 cm), corresponding to doses of 7.86, 15.72, 31.45, 62.89, and 125.79 µg/cm ^2^ . The dishes were swirled gently until the solutions formed a thin film. After the solutions evaporated for 1 min, glass loops (8.8 cm i.d. × 6.0 cm) coated with polytetrafluoroethylene were set on the interior walls of the Petri dishes to prevent insects from climbing upward. Thirty adult *S. zeamais* were placed into each Petri dish. The dishes were covered with a ventilated net lid to prevent insects from flying away. Each treatment was replicated three times, with acetone as control. The dishes were placed in a constant-temperature incubator (temperature, 27°C to 29°C; relative humidity, 70% to 80%). Mortalities were measured at 24, 48, and 72 hr after treatment. Test insects were considered dead if appendages did not move when prodded with a pin.

### Statistical analysis

Percentage repellency (PR) was calculated as follows:

$\rm PR = [(Nc-Nt)/(Nc+Nt)]\times100$

where Nc is the number of insects on the untreated area after the exposure interval, and Nt is the number of insects on the treated area after the exposure interval. Results are presented as the mean of PR ± SE. Mean PRs were assigned to repellency classes ( Malik and Mujtaba 1984 ) from 0 to V, where class 0 < 0.1%, class I = 0.1% to 20%, class II = 20.01% to 40%, class III = 40.01% to 60%, class IV = 60.01% to 80%, and class V = 80.01% to 100%. The mean PRs of each dose were compared and separated by Duncan's new multiple range tests with a significance level at α = 0.05.

The percentage mortalities were determined in contact toxicity examination and corrected using Abbott’s formula ( Abbott 1925 ). Treatment means were compared by Duncan’s new multiple range tests with the significance level at α = 0.05. Data were transformed to arcsine square-root values for analysis of variance (ANOVA). The relationship between mortality and concentration was modeled by the DPS program ( Tang and Feng 2002 ). Means (±SE) of untransformed data are presented.

## Results

### Effects of solvents on extraction yield from *Illicium verum*

The highest extraction yield from *I. verum* was obtained with MA, followed by EA and PE ( Table 1 ). The polarity values of MA, EA, and PE were 5.1, 4.4, and 0.0, respectively. The extraction yields were dependent on the similarity of the chemical characteristics of the solvent and polarity of the plant material. The result indicated that the compounds present in *I. verum* were mostly of high polarity.

**Table 1:**
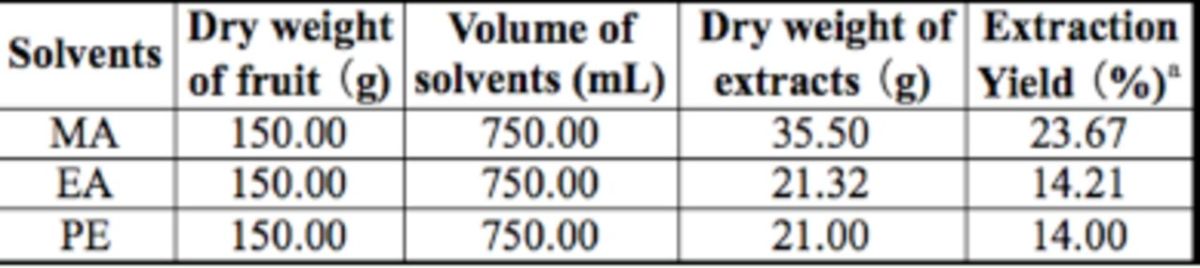
Extraction yields of *Illicium verum* using three types of organic solvents (%).

^*a*^ Extraction Yield = (Dry weight of extract / Dry weight of star anise fruit) × 100.

**Figure 1.**
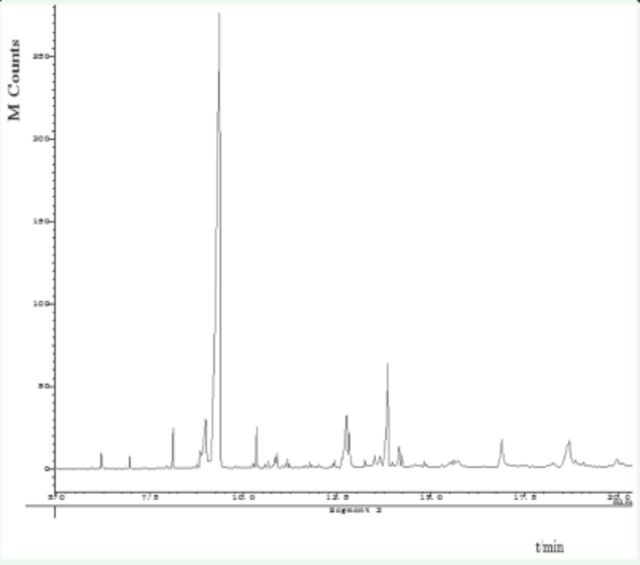
Total ion current chromatogram of compounds from *Illicium verum* by methyl alcohol extraction. High quality figures are available online.

**Figure 2.**
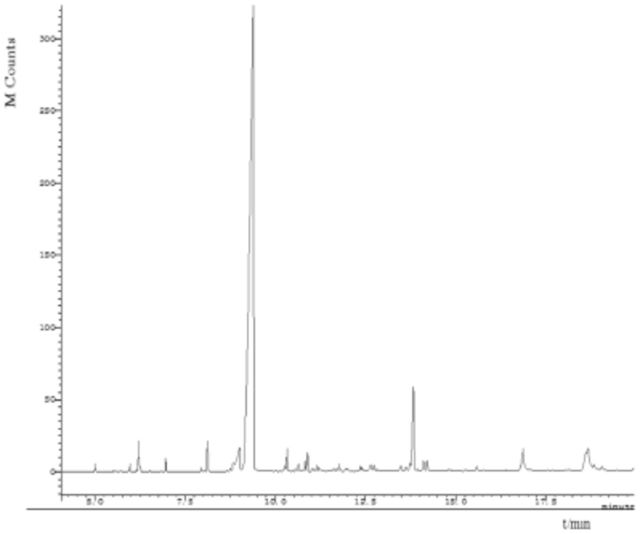
Total ion current chromatogram of compounds from *Illicium verum* by ethyl acetate extraction. High quality figures are available online.

**Figure 3.**
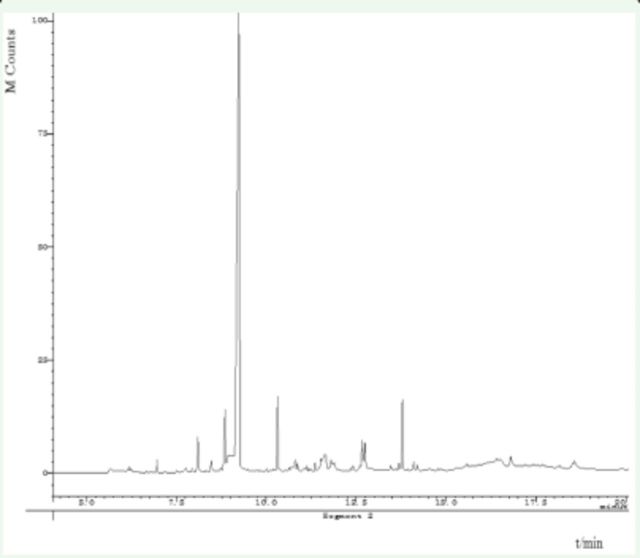
Total ion current chromatogram of compounds from *Illicium verum* by petroleum ether extraction. High quality figures are available online.

Results of GC-MS analysis showed that compositions in the three extracts were almost the same ( Figures 1–3 ). However, some minor components were different. For example, the MA extract contained more 4-ethyl benzaldehyde (3.4%) and 1-(4-methoxyphenyl)-2-propanone (3.7%); the EA extract contained more 1-(3-methyl-2-butenoxy)-4-(1-propenyl) benzene (6.2%), cis-3,5-dimethoxy-β-methyl-β-nitrostyrene (4.2%), hexadecanoic acid (4.1%), and Benzyl alcohol(4.0%); whereas the PE extract contained more 1-(3-methyl-2-butenoxy)-4-(1-propenyl) benzene (4.7%) and benzyl alcohol (2.8%).

### Chemical composition of extracts

Forty-four compounds from MA, EA, and PE extracts were separated and identified by GC-MS ( Table 2 ), representing 70.5%, 82.9%, and 92.7%, respectively, of the whole composition of the extracts. The most abundant component was trans-anethole.

**Table 2:**
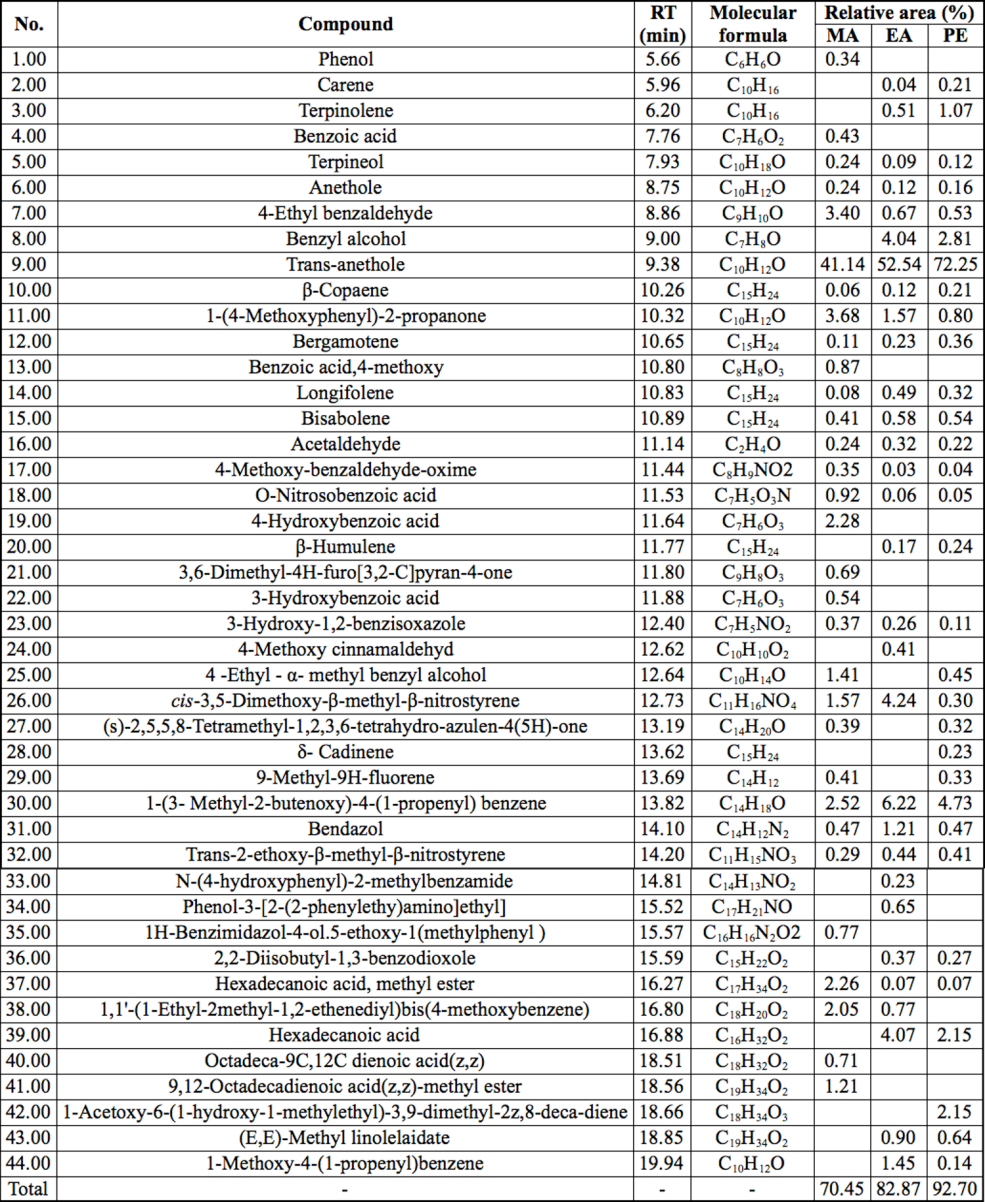
Chemical composition of extracts from *Illicium verum* dried fruit using three types of organic solvents.

### Biological activities of the extracts from *I. verum* against *S. zeamais* adults Repellency activity.

The repellent effects to *S. zeamais* adults increased with increasing dose ( Table 3 ). At the highest dose (125.79 µg/cm ^2^ ), EA had the highest average percentage repellency value, followed by PE and MA. The repellency of an extract at a dose declined as the exposure time was prolonged. This result may be attributed to solution volatilization that decreases extract concentration. In addition, the extracts from *I. verum* by three solvents to *S. zeamais* adults showed a certain paralysis effect.

**Table 3:**
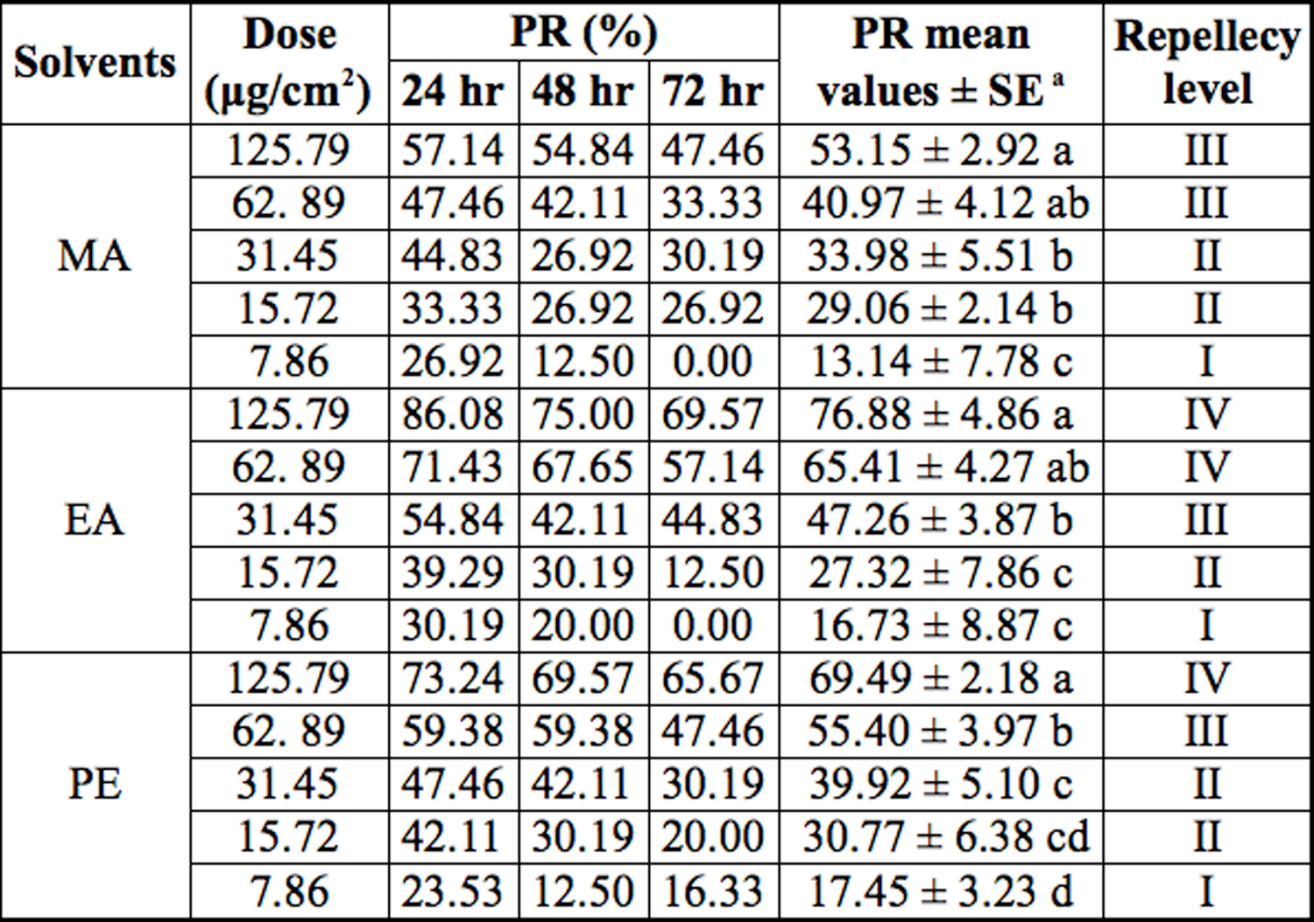
Percentage repellency (PR) of *Illicium verum* fruit extracts against *Sitophilus zeamais* adults.

^a^ The data marked with different small letters in the same column mean significant difference ( *P* < 0.05) based on Duncan's new multiple range test.

### Contact toxicity.

The extracts from *I. verum* by the three solvents showed strong contact toxicity against *S. zeamais* adults. The contact activities of the extracts by the three solvents increased with increasing treatment doses ( Table 4 ). With the highest dose of 125.79 µg/cm ^2^ at 72 hr after treatment, the mortality of *S. zeamais* adults was highest for EA, followed by PE and MA. No mortality was observed in each control. For a solvent extract at lower doses, contact killing activity increased as the processing time was prolonged. However, at higher doses, the prolonged treatment time did not translate to an increase in contact toxicity.

**Table 4:**
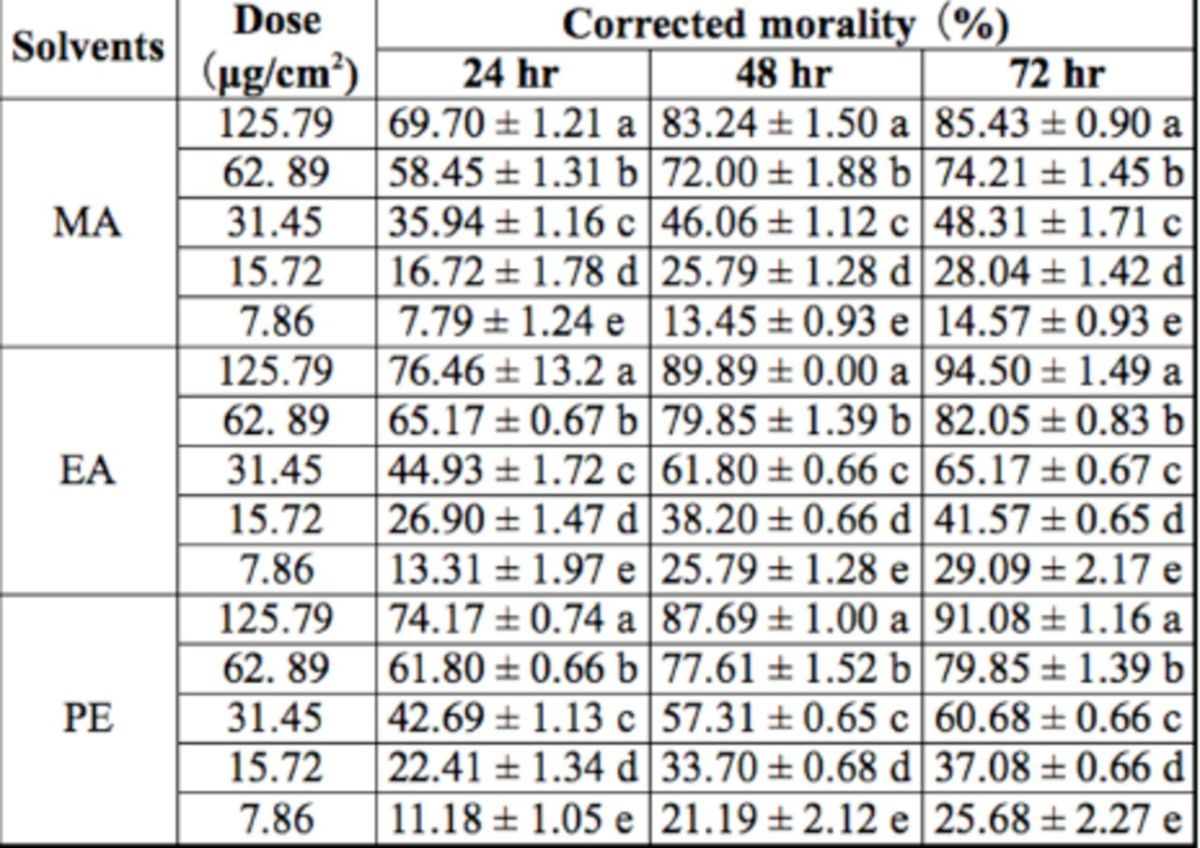
Contact toxicity of *Illicium verum* fruit extracts against *Sitophilus zeamais* adults

^a^ The dose is the weight of drug residue per unit area of Petri dishes. The data marked with different small letters in the same column mean significant difference ( *P* < 0.05) based on Duncan's new multiple range test.

The regression equations and LD _50_ ( Table 5 ) for the contact toxicities of the three extracts against *S. zeamais* adults were obtained by linear regression analysis of the correlation between the treatment doses and the arcsine square-root values of average mortalities. The contact toxicities of the extracts by all three types of solvent were enhanced with increasing treatment doses. The results indicated that EA extract had the highest contact toxicity, followed by PE extract, and finally MA extract.

**Table 5:**
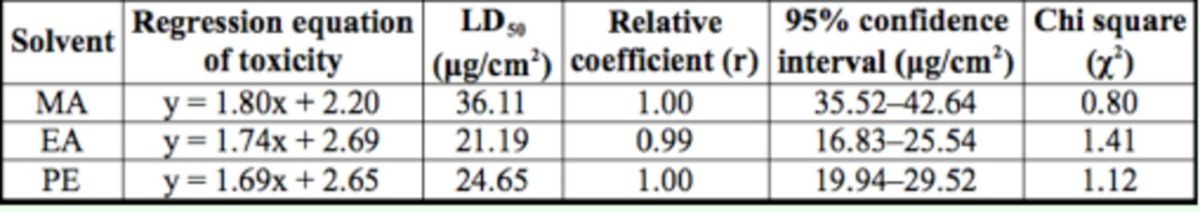
Regression analysis of contact toxicities of *Illicium verum* fruit extracts against *Sitophilus zeamais* adults at 72 hr after treatment.

## Discussion

In the current study, according to the extraction yields, yields of trans-anethole from *I. verum* in MA, EA, and PE solvents were 9.7%, 7.5%, and 10.1%, respectively, which are higher than those obtained by hydrodistillation. Moreover, the extracts obtained from MA contained more high-polar materials, whereas the extracts obtained from PE contained more non-polar materials. The polarity of the material is related to its size and molecular structure, and the polarity of target active components should be assessed to ensure that the appropriate solvent can be selected to separate them. The toxicity and reactivity of solvents with target components should also be considered.

Some minor compounds in the three extracts of *I. verum* have antibacterial activities. For example, benzyl alcohol is used as a preservative in skincare products because it is less irritating to the skin than other preservatives ( Mashayekhi et al. 2011 ). These different minor constituents provide the possibility for different applications and targeted development.

The repellency of the extracts against *S. zeamais* adults increased with increasing dose. The repellency of the extracts from the three solvents to *S. zeamais* adults was in the order of EA extract > PE extract > MA extract ( Table 3 ). The oils isolated from *Cupressus sempervirens* and *Eucalyptus saligna* are repellent against *S. zeamais* ( Tapondjou et al. 2005 ). Repellency is crucial in protecting stored products from pests ( Luz et al. 2009 ; Ukeh et al. 2009 ). In recent years, commercial repellents have developed rapidly. The current study suggests that the extracts from *I. verum* can be developed as a promising *S. zeamais* repellent applied on stored grain.

According to the mortalities and the LD50 values of MA, EA, and PE extracts to *S. zeamais* adults assayed in the current study, the EA extract had the highest contact toxicity to *S. zeamais.* Although the content of active constituent (trans-anethole) in the EA extract was the least among the three extracts, this extract showed the highest repellency and contact toxicity. This conflicting observation indicates the presence of other active constituents in the EA extract. Therefore, further studies on other insecticidal active constituents in the EA extract against *S. zeamais* and other economically important pests need to be undertaken.

Natural products can be used by small-scale farmers to protect stored grains from insect infestation. Several aromatic plants, such as *Lamiaceae,* have been used in developing countries to protect stored grain and legumes from pests ( Ngamo et al. 2007 ; Li et al. 2013 ). In West Africa, farmers traditionally introduce *Hyptis spicigera* Lamarck leaves in their granaries to protect cowpea seeds against bruchids damages ( Sanon et al. 2006 ). China has a long and rich tradition of using vegetable matter for pest control. For example, toosendanin, a good botanical insecticide, was derived from the bark of Chinaberry and is used against a broad spectrum of fruit and vegetable pests in China. *Illicium verum* is a potential grain protectant ( Ho et al. 1995 ). In the present study, the extracts from *I. verum* exhibited pest-combating activities, including both repellency and toxicity. We conclude that the *I. verum* fruit-derived materials might be useful products for managing populations of stored-product pests. *Illicium verum* has been widely cultivated in Guangxi, Yunnan, Guangdong, Fujian, and Guizhou Provinces of China. In addition, the planting area and output of China's *I. verum* account for > 80% of the planting area and output worldwide. Thus, *I. verum* is readily available in China. In integrated stored-product protection, the coarse extracts of *I. verum* may be directly used for pest prevention, repelling pests from foods, and pest control by its toxic compounds.

In recent years, considerable effort has been exerted on the potential of plant extracts or phytochemicals as sources of commercial insect-control agents or as new leads for designing target-specific molecules ( Ateyyat et al. 2009 ; Gaikwad et al. 2010 ; Li et al. 2011 ). Previous studies found that *I. verum* dried fruit contains 8% to 12% essential oil. A total of 49 compounds were separated and identified in the essential oil, including trans-anethole (81.4%), limonene (6.5%), chavicol (2.1%), and anisaldehyde (1.8%) ( Gholivand et al. 2009 ). Our future studies will focus on isolating and purifying biologically active constituents derived from the EA extract of *I. verum* to select the compound with high biological activity and relatively simple structure for the development of a new type of repellent or insecticide. Moreover, an in-depth study on the relationship between the chemical composition and insecticidal activity of the extracts need to be performed with a biological activity tracking method to provide a clearer understanding of which compounds were responsible for the extracts’ bioactivities.

## Abbreviations

EA: ethyl acetate
GC-MS: gas chromatography-mass spectrometry
MA: methyl alcohol
PE: petroleum ether
RT: retention time
